# Association Between Childhood Behavioral Problems and Insomnia Symptoms in Adulthood

**DOI:** 10.1001/jamanetworkopen.2019.10861

**Published:** 2019-09-06

**Authors:** Yohannes Adama Melaku, Sarah Appleton, Amy C. Reynolds, Alexander M. Sweetman, David J. Stevens, Leon Lack, Robert Adams

**Affiliations:** 1 Adelaide Institute for Sleep Health, College of Medicine and Public Health, Flinders University, Adelaide, South Australia, Australia; 2 The Health Observatory, Discipline of Medicine, The Queen Elizabeth Hospital Campus, University of Adelaide, Woodville, South Australia, Australia; 3 Freemason’s Centre for Men’s Health, Discipline of Medicine, The University of Adelaide, Adelaide, South Australia, Australia; 4 The Appleton Institute, The University of Central Queensland, Wayville, South Australia, Australia; 5 School of Health, Medical and Applied Sciences, The University of Central Queensland Adelaide Campus, Wayville, South Australia, Australia; 6 College of Education, Psychology, and Social Work, Flinders University of South Australia, Adelaide, South Australia, Australia

## Abstract

**Importance:**

Life-course determinants of insomnia, particularly the long-term association of childhood behavioral problems with insomnia later in life, are unknown. As childhood behaviors are measurable and potentially modifiable, understanding their associations with insomnia symptoms may provide novel insights into early intervention strategies to reduce the burden.

**Objective:**

To investigate the association between behavioral problems at 5, 10, and 16 years of age and self-reported insomnia symptoms at 42 years of age.

**Design, Setting, and Participants:**

This cohort study used data from the United Kingdom 1970 Birth Cohort Study, an ongoing large-scale follow-up study. Participants were followed up from birth (1970) to age 42 years (2012). Missing data were imputed via multiple imputation. Statistical analysis was performed from February 1 to July 15, 2019.

**Exposures:**

Behavior measured at 5, 10, and 16 years of age using the Rutter Behavioral Scale (RBS). Children’s behavior was classified as normal (≤80th percentile), moderate behavioral problems (>80th to ≤95th percentile), and severe behavioral problems (>95th percentile) based on their RBS score.

**Main Outcomes and Measures:**

Self-reported difficulties initiating or maintaining sleep (DIMS) were collected using a self-administered questionnaire at 42 years of age. Log-binomial logistic regression, adjusted for several potential confounders, was used to estimate the association of childhood behavioral problems with insomnia symptoms in adulthood. Sensitivity analyses were conducted to check robustness of the findings.

**Results:**

Participants were followed up from a baseline age of 5 years (n = 8050; 3854 boys and 4196 girls), 10 years (n = 9090; 4365 boys and 4725 girls), or 16 years (n = 7653; 3575 boys and 4078 girls) until age 42 years. There was a 39% higher risk of DIMS (odds ratio [OR], 1.39; 95% CI, 1.04-1.84; *P* = .06 for trend) for participants with severe behavioral problems at 5 years of age compared with those with a normal RBS score. The odds of DIMS plus not feeling rested on waking (DIMS plus) in participants with severe behavioral problems at 5 years of age were 29% higher (odds ratio, 1.29; 95% CI, 0.97-1.70; *P* = .14 for trend) than participants with a normal RBS score, although this result was not statistically significant. Moderate and severe behavioral problems at 16 years of age were positively associated with DIMS and DIMS plus (moderate: OR, 1.28; 95% CI, 1.07-1.52; severe: OR, 1.67; 95% CI, 1.22-2.30; *P* < .001 for trend) and DIMS plus (moderate: OR, 1.32; 95% CI, 1.11-1.56; severe: OR, 1.47; 95% CI, 1.09-1.98; *P* < .001 for trend). Externalizing behavioral problems at 5 and 10 years of age were positively associated with insomnia symptoms at 42 years of age.

**Conclusions and Relevance:**

This study is the first to show associations of early-life behavioral problems, particularly early- and middle-childhood externalizing problems, with insomnia symptoms in adulthood. These findings underline the importance of addressing insomnia from a life-course perspective and considering the benefits of early behavioral intervention to sleep health.

## Key Points

**Question:**

Are childhood behavioral problems associated with self-reported insomnia symptoms in adulthood?

**Findings:**

In this population-based cohort study, there was a significant association between childhood behavioral problems measured using the Rutter Behavioral Scale at 5, 10, and 16 years of age and self-reported insomnia symptoms at 42 years of age.

**Meaning:**

There was an increased risk of self-reported insomnia symptoms at 42 years of age among those who had moderate and severe behavioral problems during childhood, compared with those without behavioral problems during childhood.

## Teaser

This cohort study investigates the association between behavioral problems at 5, 10, and 16 years of age and self-reported insomnia symptoms at 42 years of age.

## Introduction

Insomnia is the most commonly reported sleep disorder in adults.^1^ Insomnia comprises difficulties initiating sleep (DIS), difficulties maintaining sleep (DMS), and early morning awakening, with associated daytime functional impairment.^2,3^ The prevalence of insomnia symptoms in the general population is estimated to be approximately 30%,^4^ with incidence ranging from 6% to 30%.^5,6^ Chronic insomnia is associated with an increased risk of noncommunicable diseases, psychiatric morbidity,^7,8,9^ and low quality of life.^10^ The burden of insomnia, in terms of health consequences^11^ and economic cost,^12,13^ is high and increasing.^14^ For instance, sleep disorders, including insomnia, in the United Kingdom cost an estimated $50 billion economic loss annually.^15^

Risk factors for insomnia are multifaceted and include sociodemographics,^16^ behavioral and environmental factors,^17,18^ psychiatric^19^ and substance use disorders,^20^ medical conditions,^21^ and genetics.^22^ Although these risk factors are relatively well recognized in children and adults, evidence on life-course factors and insomnia symptoms in adulthood is sparse.^23,24,25^ Although no studies to date, to our knowledge, have considered the association between childhood behaviors and subsequent symptoms of insomnia in adulthood from a life-course perspective, there are indications from existing literature that such studies would be of value. Externalizing behaviors (eg, fidgeting, destroying property, fighting, bullying, irritability, and restlessness) during childhood are associated with an increased risk of mental health problems in adulthood.^26^ The association between externalizing behaviors and insomnia symptoms is unknown; however, one study has shown that childhood sleep disorders were positively associated with adult sleep problems.^25^ In addition, childhood adversities were negatively associated with adult sleep quality.^27^ An association between childhood behavioral problems (CBPs) and subsequent sleep problems in the childhood and adolescent period has also been reported,^28,29^ but it is unknown if an association with poor sleep persists into adulthood. If behavioral problems during childhood are associated with insomnia symptoms in adulthood, managing CBPs not only has immediate benefits but may have life-course benefits through reducing the incidence and growing burden of sleep health problems.^30^ Using the 1970 UK Birth Cohort Study (BCS70),^31^ we aimed to assess the association between childhood and adolescent behavioral problems and self-reported insomnia symptoms at 42 years of age.

## Methods

### Data Source, Study Design, and Population

Details of the BCS70 are published elsewhere.^31^ In brief, the BCS70 is an ongoing cohort study that began in 1970 and covers residents across the United Kingdom. The BCS70 includes data on social, behavioral, and health conditions, collected by using self-administered and face-to-face interview questionnaires, computer-assisted telephone interviews, and medical records and examinations. To date, there have been 10 waves of data collection (birth and at 5, 10, 16, 26, 30, 34, 38, 42, and 46 years of age). The cohort began with data on 16 571 babies born in a single week. The subsequent follow-ups included 13 071 children at 5 years, 14 874 children at 10 years, and 11 621 children at 16 years.^31^ At 42 years of age, 12 198 eligible participants (those alive and not yet lost to follow-up) were invited to be interviewed; 9841 participants (80.7%) responded.^31,32,33^ In this study, we used data collected at 5, 10, 16, and 42 years of age. The sample sizes used in analyses to determine the association of childhood behavior with insomnia symptoms at 42 years of age were 8050 at 5 years, 9090 at 10 years, and 7653 at 16 years. The attrition rate was 24.7% from 5 to 42 years of age, 33.8% from 10 to 42 years of age, and 15.3% from 16 to 42 years of age (eFigure 1 in the Supplement). This study did not require ethics approval or informed consent from the participants because we used publicly available secondary data. However, approval has been sought from both institutional review and internal ethical review boards, and participants’ informed consent has been obtained, for the BCS70 follow-up surveys. Details are publicly available on the BCS70 website.^34^ This study followed the Strengthening the Reporting of Observational Studies in Epidemiology (STROBE) reporting guidelines.

### Exposure Assessment

Behavior was measured using the 19-item Rutter Behavioral Scale (RBS)^35,36^ completed by parents when the child was 5, 10, and 16 years of age. At 5 and 16 years, 3 responses (0 = does not apply; 1 = applies somewhat; and 2 = certainly applies) were summed to give an overall score ranging from 0 to 38. At 10 years, a visual analog scale system that ranges from 0 (does not apply) to 100 (certainly applies) was used. We categorized the RBS score as indicating normal behavior (≤80th percentile), moderate behavioral problems (>80th to ≤95th percentile), and severe behavioral problems (>95th percentile).^26,37^ We performed principal component analysis to identify the most relevant behavioral elements^38^ of the RBS associated with insomnia symptoms at 42 years. Rutter Behavioral Scale questions are provided in the eAppendix in the Supplement.

### Outcome Assessment

At 42 years of age, data were collected on the following 4 sleep parameters using a self-administered questionnaire: duration of sleep, DIS, DMS, and not feeling rested on waking. Questions used to collect the insomnia sleep symptoms and the responses are shown in the eAppendix in the Supplement. We defined the following 4 self-reported insomnia symptoms in the current analysis (2 single and 2 combinations of symptoms): DIS (not able to fall asleep within 30 minutes), DMS, difficulties initiating or maintaining sleep (DIMS), and DIMS plus not feeling rested on waking (DIMS plus). These symptoms are included in insomnia diagnostic criteria^2,3,39^ and commonly used in previous literature.^16^

### Covariates

A series of potential confounders were selected a priori based on a direct acyclic graph (eFigure 2 in the Supplement).^40,41^ Details on assessment and categorization of covariates that were included in this analysis are provided in the eAppendix in the Supplement. In brief, data on sex, parent-reported childhood sleep difficulty, and parents’ educational status and social class were collected at 5, 10, and 16 years of age. At 42 years of age, data on sociodemographic factors, including marital status, social class, and educational level, were collected. Behavioral factors, including smoking, alcohol consumption, and physical activity, were also assessed. Height and weight were collected at 10, 16, 26, 29, 33, 38, and 42 years of age, and body mass index (BMI) was calculated (as weight in kilograms divided by height in meters squared).^31^ At 42 years, mental health was assessed using the Warwick-Edinburgh Mental Well-being Scale.^42^ Perceived health status was determined by interview. The number of prevalent self-reported noncommunicable diseases (hypertension, diabetes, cancer, asthma, and migraine) was determined.

### Statistical Analysis

Statistical analysis was performed from February 1 to July 15, 2019. Principal component analysis was used to identify behavioral patterns within the RBS score responses and calculate factor loadings of RBS questions at 5, 10, and 16 years. We used eigenvalue (≥1.5), scree plot, and interpretability^43^ to determine the number of retained factors, followed by varimax rotation^44^ to maximize interpretation of included factors. Quintiles (quintile 1 [Q1], lowest; and quintile 5 [Q5], highest) of the factor scores for each identified behavioral pattern were constructed using the total sample. We used group-based trajectory modeling with a quadratic function to determine the trajectories of BMI.^45^

To determine the association of early childhood (5 years of age), middle childhood (10 years of age), and adolescent (16 years of age) behavioral problems in the RBS with insomnia symptoms at 42 years, we used log-binomial logistic regression and determined odds ratios (ORs) and 95% CIs. Two models were developed: model 1 was adjusted for sex, parent’s social class and educational level, marital status, educational status, and social class; model 2 was additionally adjusted for physical activity level and BMI trajectory (from 10 to 42 years), perceived health status, and number of noncommunicable diseases. Childhood behavioral problems may affect childhood sleep,^28,29^ as well as smoking,^46^ alcohol consumption,^47^ and mental well-being^26^ during adulthood. Because these factors may mediate an association between CBPs and insomnia, we did not adjust for them in our main analysis. However, we conducted secondary analyses in which these factors were considered as confounders. In addition, we performed a mediation analysis^28,48^ to examine the intermediary effect of early-life sleep difficulty^25,28^ and adulthood mental well-being.^26^ The trend of association was assessed by including the categories of RBS (1 = normal, 2 = moderate, and 3 = severe) as continuous variables in the model.

We estimated the association of stability in moderate and severe behavioral problems at the 5-year and 16-year assessments with insomnia symptoms at 42 years of age, adjusting for parents’ sociodemographic factors at 16 years of age and covariates at 42 years of age (eAppendix in the Supplement). Missing data ranged from 0% (sex and BMI trajectory) to 38.8% (parents’ social class at 16 years of age) and were imputed using a multiple imputation chained equation with 30 imputations. All model variables were included in our imputation.^49^ All findings are presented based on multiply imputed data unless otherwise indicated. A sensitivity analysis was conducted using complete-case data. We also defined the outcome (insomnia symptom) by combining DIMS and 1 of the following daytime symptoms of insomnia: tiredness, irritability, depression, and nervousness (eAppendix in the Supplement). Statistical analyses were performed using Stata, version 15.1 (StataCorp). All *P* values were from 2-sided tests and results were deemed statistically significant at *P* < .05.

## Results

### Participants’ Characteristics

Table 1 shows the characteristics of participants from the 3 baseline age samples (full and multiply imputed data). A total of 2040 of 8050 participants (25.3% [multiply imputed data, 25.5%]) at 5 years, 1449 of 9090 participants (15.9% [multiply imputed data, 17.0%]) at 10 years, and 1215 of 7653 participants (15.9% [multiply imputed data, 31.0%]) at 16 years had parent-reported difficulty of sleep.

**Table 1.** Characteristics of Participants at 5, 10, 16, and 42 Years of Age for the 5, 10, and 16 Years of Age Samples in the UK 1970 Birth Cohort Study

Variable	At 5 y		At 10 y		At 16 y	
	Full Data (n = 8050), No. (%)	Multiply Imputed Data, %	Full Data (n = 9090), No. (%)	Multiply Imputed Data, %	Full Data (n = 7653), No. (%)	Multiply Imputed Data, %
Male sex	3854 (47.9)	0	4365 (48.0)	0	3575 (46.7)	0
Behavior at 5, 10, and 16 y						
Normal	6289 (78.1)	81.9	6440 (70.8)	79.4	4470 (58.4)	82.4
Moderate behavioral problem	1070 (13.3)	14.0	1207 (13.3)	15.2	707 (9.2)	13.2
Severe behavioral problem	302 (3.8)	4.1	402 (4.4)	5.4	219 (2.9)	4.4
Missing	389 (4.8)	0	1041 (11.5)	0	2257 (29.5)	0
Sleep difficulty at 5, 10, and 16 y						
Yes	2040 (25.3)	25.5	1449 (15.9)	17.0	1215 (15.9)	31.0
Missing	47 (0.6)	0	564 (6.2)	0	3735 (48.8)	0
Father or mother’s social class at 5, 10, and 16 y^a^						
SC I and II	2239 (27.8)	28.8	2511 (27.6)	32.2	1782 (23.3)	36.3
SC III NM and III M	4187 (52.0)	54.2	4113 (45.2)	53.1	2301 (30.1)	50.2
SC IV and V	1302 (16.2)	17.0	1119 (12.3)	14.7	508 (6.6)	11.5
Student	NA	NA	NA	NA	89 (1.2)	2.1
Missing	322 (4.0)	0	1347 (14.8)	0	2973 (38.8)	0
Mother’s social class at 10 and 16 y						
SC I and II	NA	NA	2539 (27.9)	32.6	870 (11.4)	17.4
SC III NM and III M	NA	NA	4039 (44.4)	52.5	1812 (23.7)	37.0
SC IV and V	NA	NA	1130 (12.4)	14.9	1079 (14.1)	21.9
Student	NA	NA	NA	NA	1141 (14.9)	23.7
Missing	NA	NA	1382 (15.2)	0	2751 (35.9)	0
Father or mother’s educational level at 5, 10, and 16 y						
No qualification	3265 (40.6)	45.4	2700 (29.7)	34.9	1155 (15.1)	25.8
Vocational or certificate	2711 (33.7)	37.3	3413 (37.5)	44.0	2502 (32.7)	52.5
Degree and above	1143 (14.2)	15.4	948 (10.4)	12.0	1036 (13.5)	20.2
Other	145 (1.8)	1.9	710 (7.8)	9.0	75 (1.0)	1.4
Missing	786 (9.8)	0	1319 (14.5)	0	2885 (37.7)	0
Mother’s educational level at 5 and 10 y						
No qualification	4048 (50.3)	52.3	3988 (43.9)	49.3	NA	NA
Vocational or certificate	3363 (41.8)	43.8	3454 (38.0)	42.8	NA	NA
Degree and above	229 (2.8)	3.0	245 (2.7)	3.0	NA	NA
Other	74 (0.9)	1.0	395 (4.3)	4.9	NA	NA
Missing	336 (4.2)	0	1008 (11.1)	0	NA	NA
Marital status						
Single	1925 (23.9)	23.9	2184 (24.0)	24.1	1779 (23.2)	23.3
Married or partnered	5047 (62.7)	62.8	5668 (62.4)	62.4	4843 (63.3)	63.6
Divorced, separated, or widowed	1067 (13.3)	13.3	1225 (13.5)	13.5	1021 (13.3)	13.3
Missing	11 (0.1)	0	13 (0.1)	0	10 (0.1)	0
Social class						
SC I and II	3441 (42.7)	48.8	3887 (42.8)	49.0	3370 (44.0)	50.3
SC III NM and III M	2422 (30.1)	36.1	2709 (29.8)	35.8	2261 (29.5)	35.2
SC IV and V	943 (11.7)	14.7	1070 (11.8)	14.8	877 (11.5)	14.1
Other	22 (0.3)	0.4	27 (0.3)	0.4	23 (0.3)	0.4
Missing	1222 (15.2)	0	1397 (15.4)	0	1122 (14.7)	0
Educational qualification						
None	2119 (26.3)	26.3	2412 (26.5)	26.6	1933 (25.3)	25.3
NVQ level 1	1565 (19.4)	19.5	1742 (19.2)	19.2	1480 (19.3)	19.4
NVQ level 2	1492 (18.5)	18.5	1697 (18.7)	18.7	1408 (18.4)	18.4
NVQ level 4	1224 (15.2)	15.2	1386 (15.2)	15.3	1194 (15.6)	15.6
NVQ level 4	1367 (17.0)	17.0	1525 (16.8)	16.8	1335 (17.4)	17.5
NVQ level 5	279 (3.5)	3.5	321 (3.5)	3.5	298 (3.9)	3.9
Missing	4 (0.0)	0	7 (0.1)	0	5 (0.1)	0
Smoking						
Nonsmoker	3726 (46.3)	46.4	4176 (45.9)	46.1	3628 (47.4)	47.5
Ex-smoker	2247 (27.9)	28.0	2546 (28.0)	28.1	2136 (27.9)	28.0
Current smoker	2045 (25.4)	25.6	2333 (25.7)	25.8	1868 (24.4)	24.5
Missing	32 (0.4)	0	35 (0.4)	0	21 (0.3)	0
Alcoholic consumption						
Never	657 (8.2)	9.4	759 (8.3)	9.6	628 (8.2)	9.3
Monthly or less	1425 (17.7)	20.3	1593 (17.5)	20.1	1326 (17.3)	19.7
2-4 Times/mo	1665 (20.7)	23.5	1888 (20.8)	23.7	1628 (21.3)	24.1
2-3 Times/wk	2150 (26.7)	30.3	2409 (26.5)	30.2	2060 (26.9)	30.5
≥4 Times/wk	1168 (14.5)	16.5	1309 (14.4)	16.4	1108 (14.5)	16.4
Missing	985 (12.2)	0	1132 (12.5)	0	903 (11.8)	0
Physical activity in the last 12 mo						
Not in the last 12 mo	354 (4.4)	5.1	411 (4.5)	5.2	336 (4.4)	5.0
Less often	1208 (15.0)	17.1	1351 (14.9)	17.0	1150 (15.0)	17.1
2-3 Times/mo	1096 (13.6)	15.4	1245 (13.7)	15.5	1058 (13.8)	15.6
1 Time/wk	1257 (15.6)	17.6	1392 (15.3)	17.3	1187 (15.5)	17.4
2-3 Days/wk	1676 (20.8)	23.4	1877 (20.6)	23.2	1623 (21.2)	23.6
4-5 Days/wk	793 (9.9)	11.0	904 (9.9)	11.1	771 (10.1)	11.2
Every day	755 (9.4)	10.4	860 (9.5)	10.6	695 (9.1)	10.1
Missing	911 (11.3)	0	1050 (11.6)	0	833 (10.9)	0
BMI trajectory						
Increasing to normal	4461 (55.4)	NA	5106 (56.2)	NA	4288 (56.0)	NA
Increasing to overweight	2941 (36.5)	NA	3272 (36.0)	NA	2750 (35.9)	NA
Increasing to obesity	648 (8.0)	NA	712 (7.8)	NA	615 (8.0)	NA
Mental health, WEMWBS scale score, mean (SD)	40.6 (20.1)	NA	40.5 (20.2)	NA	41.1 (19.9)	NA
Self-reported health condition						
Excellent	1744 (21.7)	21.7	1978 (21.8)	21.8	1718 (22.4)	22.5
Very good	2914 (36.2)	36.3	3296 (36.3)	36.4	2765 (36.1)	36.2
Good	2165 (26.9)	27.0	2437 (26.8)	26.9	2032 (26.6)	26.6
Fair	838 (10.4)	10.5	941 (10.4)	10.4	806 (10.5)	10.6
Poor	358 (4.4)	4.5	401 (4.4)	4.4	307 (4.0)	4.0
Missing	31 (0.4)	0	37 (0.4)	0	25 (0.3)	0
NCDs, No.						
None	5545 (68.9)	69.1	6262 (68.9)	69.0	5242 (68.5)	68.6
1	2028 (25.2)	25.3	2285 (25.1)	25.2	1951 (25.5)	25.5
≥2	456 (5.7)	5.7	521 (5.7)	5.7	446 (5.8)	5.8
Missing	21 (0.3)	0	22 (0.2)	0	14 (0.2)	0
Difficulties initiating sleep						
Yes	1499 (18.6)	21.6	1699 (18.7)	21.7	1402 (18.3)	21.1
Missing	977 (12.1)	0	1128 (12.4)	0	907 (11.9)	0
Difficulties maintaining sleep						
Yes	1583 (19.7)	22.7	1809 (19.9)	22.9	1478 (19.3)	22.1
Missing	972 (12.1)	0	1118 (12.3)	0	894 (11.7)	0
DIMS						
Yes	2251 (28.0)	33.3	2556 (28.1)	32.5	2105 (27.5)	31.5
Missing	985 (12.2)	0	1134(12.5)	0	909 (11.9)	0
DIMS plus not feeling rested on waking						
Yes	1906 (23.7)	27.3	2168 (23.9)	27.6	1784 (23.3)	26.8
Missing	974 (12.1)	0	1127 (12.4)	0	902 (11.8)	0
DIMS plus ≥1 daytime symptom						
Yes	1623 (20.2)	23.3	1852 (20.4)	23.6	1521 (19.9)	22.9
Missing	995 (12.4)	0	1144 (12.6)	0	916 (12.0)	0

Abbreviations: BMI, body mass index; DIMS, difficulties initiating or maintaining sleep; M, manual; NA, not applicable; NCD, noncommunicable disease; NM, nonmanual; NVQ, National Vocational Qualification; SC, social class; WEMWBS, Warwick-Edinburgh Mental Well-being Scale.

^a^ Social class definitions: I, professional worker; II, managerial and technical worker; III NM, nonmanual worker; III M, manual worker; IV, partly skilled worker; and V, unskilled worker.

In the sample with a baseline age of 5 years, 1925 of 8050 of the participants were single (23.9% [multiply imputed data, 23.9%]) and 2045 of 8050 (25.4% [multiply imputed data, 25.6%]) were smokers at age 42 years. Alcohol consumption 4 or more times per week was reported by 1168 of 8050 participants (14.5% [multiply imputed data, 16.5%]). A total of 755 of 8050 participants (9.4% [multiply imputed data, 10.4%]) did some form of exercise every day (Table 1). We found 3 patterns of BMI trajectories: increasing to normal, increasing to overweight, and increasing to obesity (eFigure 3 in the Supplement).

### Behavioral Patterns

Two main factors appeared from the factor analysis and could be characterized by the behavioral patterns of externalizing and internalizing behaviors (eFigure 4 in the Supplement). The externalizing pattern was characterized by lying, disobedience, bullying, taking things belonging to others, destroying belongings, fighting, restlessness, and inability to settle. The internalizing behavior pattern was characterized by worry, fearfulness, fussiness or being overly particular, solitariness (doing things on own), miserableness, tearfulness, and distress. The proportion of variances explained by these 2 patterns were 19.7% for externalizing and 8.8% for internalizing at 5 years, 24.8% for externalizing and 8.8% for internalizing at 10 years, and 24.1% for externalizing and 8.8% for internalizing at 16 years.

### Association Between CBPs and Insomnia Symptoms in Adulthood

The prevalence of insomnia symptoms at 42 years of age across RBS categories at 5, 10, and 16 years of age is depicted in eFigure 5 in the Supplement (multiply imputed data). We found, in general, an increasing trend of insomnia symptoms at 42 years across RBS categories from normal to severe, for the groups with baseline age of 5, 10, and 16 years. The prevalence of DIMS at 42 years was 33.3% for the 5-year baseline sample (2681 of 8050), 32.5% for the 10-year baseline sample (2954 of 9090), and 31.5% for the 16-year baseline sample (2411 of 7653). DIMS plus at 42 years of age was reported in 27.3% of the 5-year baseline sample (2198 of 8050), 27.6% of the 10-year baseline sample (2173 of 9090), and 26.8% of the 16-year baseline sample (2051 of 7653) (Table 1).

Model 1 showed a 41% increase in the odds of DIS in participants with severe behavioral problems at 5 years of age compared with those with normal behavior (OR, 1.41; 95% CI, 1.06-1.86; *P* = .05 for trend) (Table 2). This association was attenuated in model 2 (OR, 1.28; 95% CI, 0.95-1.72; *P* = .36 for trend). In model 1, severe behavioral problems at 5 years of age were associated with DIMS at 42 years of age (OR, 1.50; 95% CI, 1.14-1.96; *P* = .004 for trend). In the fully adjusted model (model 2), the association was slightly attenuated (OR, 1.39; 95% CI, 1.04-1.84; *P* = .06 for trend). There were increased odds of DIMS plus in participants with severe behavioral problems at 5 years of age compared with those with normal behavior (OR, 1.41; 95% CI, 1.07-1.84; *P* = .01 for trend). This association was slightly attenuated in the fully adjusted model (OR, 1.29; 95% CI, 0.97-1.70; *P* = .14 for trend).

**Table 2.** Association of Rutter Behavioral Scale Categories at 5, 10, and 16 Years of Age With Self-reported Insomnia Symptoms at 42 Years of Age in the UK 1970 Birth Cohort Study

Model^a^	Odds Ratio (95% CI)			*P* Value for Trend
	Normal Behavior	Moderate Behavioral Problems	Severe Behavioral Problems	
**Difficulties Initiating Sleep**				
At 5 y (n = 8050)				
Model 1	1 [Reference]	1.03 (0.86-1.22)	1.41 (1.06-1.86)^b^	.05
Model 2	1 [Reference]	0.96 (0.80-1.15)	1.28 (0.95-1.72)	.36
At 10 y (n = 9090)				
Model 1	1 [Reference]	1.24 (1.06-1.44)^c^	1.24 (0.97-1.58)	.006
Model 2	1 [Reference]	1.14 (0.97-1.33)	1.04 (0.80-1.36)	.28
At 16 y (n = 7653)				
Model 1	1 [Reference]	1.64 (1.35-2.01)^d^	2.17 (1.58-2.98)^d^	<.001
Model 2	1 [Reference]	1.44 (1.16-1.79)^c^	1.63 (1.17-2.28)^c^	<.001
**Difficulties Maintaining Sleep**				
At 5 y				
Model 1	1 [Reference]	1.15 (0.98-1.35)	1.24 (0.93-1.67)	.04
Model 2	1 [Reference]	1.08 (0.91-1.28)	1.13 (0.83-1.53)	.27
At 10 y				
Model 1	1 [Reference]	1.21 (1.03-1.42)^b^	1.19 (0.94-1.51)	.01
Model 2	1 [Reference]	1.11 (0.94-1.31)	1.00 (0.78-1.30)	.46
At 16 y				
Model 1	1 [Reference]	1.40 (1.16-1.69)^c^	2.15 (1.56-2.97)^d^	<.001
Model 2	1 [Reference]	1.23 (1.00-1.50)^b^	1.63 (1.15-2.30)^c^	.002
**Difficulties Initiating or Maintaining Sleep**				
At 5 y				
Model 1	1 [Reference]	1.09 (0.94-1.27)	1.50 (1.14-1.96)^c^	.004
Model 2	1 [Reference]	1.03 (0.88-1.20)	1.39 (1.04-1.84)^b^	.06
At 10 y				
Model 1	1 [Reference]	1.23 (1.07-1.41)^c^	1.30 (1.04-1.63)^b^	.001
Model 2	1 [Reference]	1.13 (0.98-1.31)	1.12 (0.88-1.44)	.10
At 16 y				
Model 1	1 [Reference]	1.45 (1.23-1.71)^d^	2.15 (1.59-2.91)^d^	<.001
Model 2	1 [Reference]	1.28 (1.07-1.52)^c^	1.67 (1.22-2.30)^c^	<.001
**Difficulties Initiating or Maintaining Sleep Plus**				
At 5 y				
Model 1	1 [Reference]	1.09 (0.93-1.29)	1.41 (1.07-1.84)^b^	.01
Model 2	1 [Reference]	1.02 (0.86-1.21)	1.29 (0.97-1.70)	.14
At 10 y				
Model 1	1 [Reference]	1.25 (1.08-1.44)^c^	1.39 (1.10-1.75)^c^	<.001
Model 2	1 [Reference]	1.15 (0.98-1.34)	1.20 (0.93-1.54)	.045
At 16 y				
Model 1	1 [Reference]	1.50 (1.27-1.77)^d^	1.96 (1.48-2.58)^d^	<.001
Model 2	1 [Reference]	1.32 (1.11-1.56)^c^	1.47 (1.09-1.98)^b^	<.001

^a^ Model 1 was adjusted for sex, parents’ social class, parents’ educational status, participants’ social class, participants’ educational status, and participants’ marital status; model 2 was additionally adjusted for physical activity, body mass index trajectory (from 10 to 42 years of age), perceived health status, and number of noncommunicable diseases.

^b^ *P* < .05.

^c^ *P* < .01.

^d^ *P* < .001.

Model 1 showed a 115% increase in odds of DMS in participants with severe behavioral problems at 16 years of age compared with those with normal behavior (OR, 2.15; 95% CI, 1.56-2.97; *P* < .001 for trend). This association was slightly attenuated in model 2 (OR, 1.63; 95% CI, 1.15-2.30; *P* = .002 for trend). In model 1, participants with severe behavioral problems at 16 years of age had an increased risk of DIMS compared with those with normal behavior (OR, 2.15; 95% CI, 1.59-2.91; *P* < .001 for trend), an increase that persisted in model 2 (OR, 1.67; 95% CI, 1.22-2.30; *P* < .001 for trend). There were increased odds of DIMS plus in participants with severe behavioral problems at 16 years of age compared with those with normal behavior (OR, 1.96; 95% CI, 1.48-2.58; *P* < .001 for trend). This association was slightly attenuated, but remained high, in the fully adjusted model (OR, 1.47; 95% CI, 1.09-1.98; *P* < .001 for trend) (Table 2).

The stability of behavioral problems across the assessment of participants 5 and 16 years of age and the association with insomnia at 42 years of age are depicted in the Figure. Compared with those who had normal RBS scores at both 5 and 16 years of age, those who had moderate or severe behavioral problems at both ages had increased odds of DIMS (OR, 1.40; 95% CI, 1.05-1.86).

**Figure.**
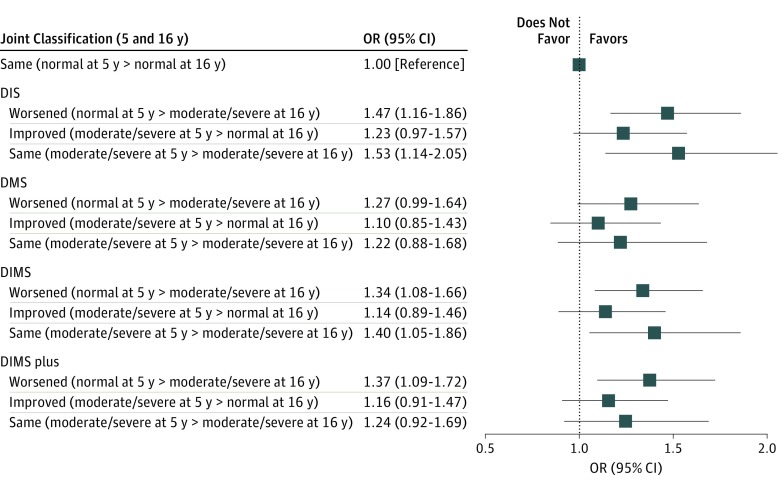
Odds Ratios (ORs) for Self-reported Insomnia Symptoms at 42 Years of Age Associated With Stability of Behavioral Problems Measured With the Rutter Behavioral Scale at 5 and 16 Years of Age Among 6462 Participants in the UK 1970 Birth Cohort Study The model was adjusted for sex, parents’ social class (16 years of age), parents’ educational level (16 years of age), participants’ social class, participants’ marital status, participants’ educational status, physical activity level, body mass index trajectory (from 10 to 42 years of age), perceived health status, and number of noncommunicable diseases. DIMS indicates difficulties initiating or maintaining sleep; DIMS plus, difficulties initiating or maintaining sleep plus not feeling rested on waking; DIS, difficulties initiating sleep; and DMS, difficulties maintaining sleep.

Approximately 16% (OR for the natural indirect effect, 1.04; 95% CI, 1.00-1.09) of the total association of childhood behavior (normal vs severe) at 5 years of age with the odds of DIMS plus at 42 years was mediated through sleep difficulty at 5 years. Mental well-being at 42 years mediated 24% (OR for the natural indirect effect, 1.04; 95% CI, 1.01-1.08) of the association of childhood behavior (normal vs sever) at 5 years and DIMS at 42 years.

Behavioral problems had a similar association trend with DIMS plus and at least 1 daytime symptom of insomnia (irritability, depression, tiredness, or nervousness) compared with the other definitions of insomnia symptoms (eTable 1 in the Supplement). Adjusting for additional covariates (childhood sleep difficulties, smoking, alcohol consumption, and mental well-being) made little or no difference to estimated associations between behavioral problems at 5, 10, and 16 years of age and insomnia symptoms (eTable 2 in the Supplement). Whereas complete-case analysis attenuated the association at 16 years, the association was inflated at 5 years (eTable 3 in the Supplement).

### Behavioral Patterns at 5, 10, and 16 Years of Age and Insomnia Symptoms at 42 Years of Age

The prevalence of insomnia symptoms showed an increasing trend across quintiles of externalizing behavior at 5 and 10 years of age. In contrast, a declining trend in the prevalence of DIS, DMS, and DIMS across the quintiles of internalizing behavior at 16 years of age was found (eFigure 5 in the Supplement).

Estimated associations between quintiles (ORs for Q1 vs Q2, Q3, Q4, and Q5) of externalizing and internalizing behavior at 5, 10, and 16 years of age and insomnia symptoms at 42 years of age are shown in Table 3 and Table 4. We found a positive association between externalizing behavior at 5 years of age and all ranges of sleep symptoms at 42 years. An inverse association of externalizing (inconsistent across insomnia symptoms) and internalizing (consistent across insomnia symptoms) behavioral problems at 16 years and insomnia symptoms at 42 years was observed.

**Table 3.** Association of Externalizing Behavioral Problems at 5, 10, and 16 Years of Age With Self-reported Insomnia Symptoms at 42 Years of Age in the UK 1970 Birth Cohort Study

Model^a^	Odds Ratio (95% CI)					*P* Value for Trend
	Quintile 1	Quintile 2	Quintile 3	Quintile 4	Quintile 5	
**Difficulties Initiating Sleep**						
At 5 y (n = 8050)						
Model 1	1 [Reference]	1.20 (0.99-1.45)	1.35 (1.11-1.64)^b^	1.25 (1.03-1.52)^c^	1.33 (1.09-1.61)^b^	.009
Model 2	1 [Reference]	1.21 (0.99-1.48)	1.34 (1.09-1.64)^b^	1.23 (1.00-1.51)^c^	1.16 (0.95-1.42)	.24
At 10 y (n = 9090)						
Model 1	1 [Reference]	1.19 (0.99-1.44)	1.16 (0.96-1.40)	1.14 (0.95-1.36)	1.33 (1.11-1.60)^b^	.01
Model 2	1 [Reference]	1.14 (0.94-1.39)	1.11 (0.91-1.35)	1.10 (0.91-1.32)	1.15 (0.95-1.40)	.27
At 16 y (n = 7653)						
Model 1	1 [Reference]	0.75 (0.61-0.93)^b^	0.64 (0.52-0.79)^b^	0.70 (0.55-0.89)^b^	0.70 (0.55-0.87)^b^	.003
Model 2	1 [Reference]	0.85 (0.68-1.06)	0.77 (0.61-0.96)^c^	0.81 (0.62-1.04)	0.82 (0.63-1.06)	.10
**Difficulties Maintaining Sleep**						
At 5 y						
Model 1	1 [Reference]	1.08 (0.89-1.31)	1.42 (1.18-1.71)^d^	1.31 (1.08-1.59)^b^	1.56 (1.29-1.89)^d^	<.001
Model 2	1 [Reference]	1.08 (0.89-1.32)	1.40 (1.16-1.69)^b^	1.27 (1.04-1.54)^c^	1.38 (1.13-1.68)^c^	.001
At 10 y						
Model 1	1 [Reference]	1.02 (0.85-1.23)	1.17 (0.97-1.42)	1.08 (0.90-1.29)	1.39 (1.16-1.68)^b^	.001
Model 2	1 [Reference]	0.98 (0.81-1.18)	1.13 (0.93-1.36)	1.03 (0.86-1.24)	1.21 (1.00-1.47)^c^	.04
At 16 y						
Model 1	1 [Reference]	0.71 (0.59-0.86)^d^	0.63 (0.52-0.77)^d^	0.66 (0.53-0.81)^d^	0.63 (0.51-0.77)^d^	<.001
Model 2	1 [Reference]	0.80 (0.65-0.98)^c^	0.75 (0.61-0.93)^b^	0.75 (0.60-0.93)^b^	0.73 (0.59-0.91)^b^	.006
**Difficulties Initiating or Maintaining Sleep**						
At 5 y						
Model 1	1 [Reference]	1.12 (0.94-1.33)	1.38 (1.17-1.62)^d^	1.30 (1.10-1.54)^b^	1.52 (1.27-1.81)^d^	<.001
Model 2	1 [Reference]	1.12 (0.94-1.34)	1.36 (1.14-1.61)^d^	1.26 (1.06-1.51)^b^	1.35 (1.12-1.62)^b^	.001
At 10 y						
Model 1	1 [Reference]	1.05 (0.89-1.25)	1.12 (0.95-1.32)	1.13 (0.96-1.34)	1.39 (1.17-1.64)^d^	<.001
Model 2	1 [Reference]	1.01 (0.85-1.20)	1.08 (0.91-1.28)	1.09 (0.92-1.30)	1.22 (1.01-1.46)^c^	.02
At 16 y						
Model 1	1 [Reference]	0.72 (0.59-0.87)^b^	0.65 (0.54-0.79)^d^	0.71 (0.59-0.86)^d^	0.65 (0.54-0.78)^d^	<.001
Model 2	1 [Reference]	0.80 (0.66-0.98)^c^	0.77 (0.63-0.94)^c^	0.81 (0.66-0.99)^c^	0.75 (0.62-0.91)^b^	.01
**Difficulties Initiating or Maintaining Sleep Plus**						
At 5 y						
Model 1	1 [Reference]	1.08 (0.91-1.30)	1.27 (1.07-1.50)^b^	1.19 (1.00-1.41)	1.48 (1.23-1.77)^d^	<.001
Model 2	1 [Reference]	1.08 (0.90-1.29)	1.24 (1.04-1.48)^c^	1.14 (0.95-1.36)	1.29 (1.06-1.56)^c^	.01
At 10 y						
Model 1	1 [Reference]	1.04 (0.87-1.24)	1.12 (0.95-1.33)	1.11 (0.94-1.32)	1.38 (1.16-1.65)^d^	<.001
Model 2	1 [Reference]	0.99 (0.83-1.19)	1.07 (0.90-1.28)	1.07 (0.89-1.28)	1.20 (1.00-1.45)^c^	.03
At 16 y						
Model 1	1 [Reference]	0.70 (0.58-0.85)^d^	0.65 (0.54-0.78)^d^	0.71 (0.57-0.89)^b^	0.67 (0.55-0.81)^d^	<.001
Model 2	1 [Reference]	0.78 (0.64-0.96)^c^	0.77 (0.63-0.94)^c^	0.81 (0.64-1.02)	0.78 (0.63-0.96)^c^	.05

^a^ Model 1 was adjusted for sex, parents’ social class, parents’ educational status, participants’ social class, participants’ educational status, and participants’ marital status; model 2 was additionally adjusted for physical activity, body mass index trajectory (from 10 to 42 years of age), perceived health status, and number of noncommunicable diseases.

^b^ *P* < .01.

^c^ *P* < .05.

^d^ *P* < .001.

**Table 4.** Association of Internalizing Behavioral Problems at 5, 10, and 16 Years of Age With Self-reported Insomnia Symptoms at 42 Years of Age in the UK 1970 Birth Cohort Study

Model^a^	Odds Ratio (95% CI)					*P* Value for Trend
	Quintile 1	Quintile 2	Quintile 3	Quintile 4	Quintile 5	
**Difficulties Initiating Sleep**						
At 5 y (n = 8050)						
Model 1	1 [Reference]	1.03 (0.85-1.23)	0.95 (0.79-1.14)	1.10 (0.92-1.32)	1.04 (0.86-1.24)	.50
Model 2	1 [Reference]	0.99 (0.82-1.20)	0.91 (0.75-1.11)	1.11 (0.92-1.34)	1.02 (0.84-1.23)	.50
At 10 y (n = 9090)						
Model 1	1 [Reference]	0.85 (0.71-1.02)	1.15 (0.96-1.36)	1.06 (0.89-1.26)	1.08 (0.91-1.28)	.08
Model 2	1 [Reference]	0.85 (0.70-1.02)	1.13 (0.95-1.35)	1.02 (0.85-1.22)	1.04 (0.87-1.25)	.24
At 16 y (n = 7653)						
Model 1	1 [Reference]	0.69 (0.57-0.84)^b^	0.63 (0.51-0.78)^b^	0.54 (0.44-0.66)^b^	0.56 (0.44-0.71)^b^	<.001
Model 2	1 [Reference]	0.75 (0.62-0.91)^c^	0.68 (0.55-0.85)^c^	0.59 (0.47-0.73)^b^	0.62 (0.49-0.81)^b^	<.001
**Difficulties Maintaining Sleep**						
At 5 y						
Model 1	1 [Reference]	0.91 (0.75-1.09)	1.03 (0.86-1.23)	1.00 (0.83-1.22)	1.03 (0.85-1.23)	.49
Model 2	1 [Reference]	0.88 (0.72-1.07)	1.00 (0.83-1.20)	1.01 (0.83-1.23)	1.01 (0.83-1.22)	.50
At 10 y						
Model 1	1 [Reference]	1.05 (0.88-1.26)	1.19 (1.00-1.42)^d^	1.11 (0.93-1.33)	1.03 (0.85-1.23)	.63
Model 2	1 [Reference]	1.06 (0.88-1.28)	1.19 (0.99-1.42)	1.08 (0.89-1.30)	0.99 (0.82-1.20)	.95
At 16 y						
Model 1	1 [Reference]	0.81 (0.67-0.98)^d^	0.78 (0.64-0.95)^d^	0.76 (0.62-0.93)^c^	0.73 (0.60-0.90)^c^	.003
Model 2	1 [Reference]	0.90 (0.74-1.09)	0.85 (0.69-1.04)	0.85 (0.69-1.04)	0.84 (0.68-1.03)	.08
**Difficulties Initiating or Maintaining Sleep**						
At 5 y						
Model 1	1 [Reference]	0.93 (0.78-1.10)	0.94 (0.80-1.11)	1.04 (0.88-1.23)	1.02 (0.86-1.21)	.46
Model 2	1 [Reference]	0.90 (0.75-1.07)	0.90 (0.76-1.08)	1.04 (0.87-1.24)	1.00 (0.84-1.20)	.48
At 10 y						
Model 1	1 [Reference]	0.95 (0.81-1.11)	1.18 (1.00-1.38)^d^	1.07 (0.92-1.25)	1.07 (0.91-1.25)	.16
Model 2	1 [Reference]	0.95 (0.81-1.12)	1.17 (0.99-1.38)	1.04 (0.88-1.23)	1.32 (0.88-1.22)	.42
At 16 y						
Model 1	1 [Reference]	0.78 (0.66-0.93)^c^	0.72 (0.60-0.86)^b^	0.66 (0.55-0.80)^b^	0.67 (0.56-0.81)^b^	<.001
Model 2	1 [Reference]	0.85 (0.71-1.02)	0.78 (0.64-0.94)^c^	0.73 (0.59-0.89)^c^	0.76 (0.62-0.92)^c^	.001
**Difficulties Initiating or Maintaining Sleep Plus**						
At 5 y						
Model 1	1 [Reference]	0.91 (0.76-1.08)	0.94 (0.79-1.12)	1.02 (0.87-1.21)	0.99 (0.83-1.18)	.59
Model 2	1 [Reference]	0.87 (0.73-1.04)	0.90 (0.75-1.08)	1.02 (0.86-1.21)	0.97 (0.81-1.16)	.64
At 10 y						
Model 1	1 [Reference]	0.96 (0.81-1.13)	1.09 (0.91-1.30)	1.02 (0.85-1.22)	1.12 (0.95-1.33)	.15
Model 2	1 [Reference]	0.96 (0.80-1.14)	1.08 (0.89-1.29)	0.98 (0.81-1.18)	1.08 (0.91-1.30)	.38
At 16 y						
Model 1	1 [Reference]	0.80 (0.67-0.96)^d^	0.70 (0.58-0.85)^b^	0.66 (0.54-0.79)^b^	0.67 (0.54-0.82)^b^	<.001
Model 2	1 [Reference]	0.88 (0.73-1.06)	0.76 (0.62-0.93)^c^	0.73 (0.60-0.88)^c^	0.76 (0.62-0.95)^d^	.001

^a^ Model 1 was adjusted for sex, parents’ social class, parents’ educational status, participants’ social class, participants’ educational status, and participants’ marital status; model 2 was additionally adjusted for physical activity, body mass index trajectory (from 10 to 42 years of age), perceived health status, and number of noncommunicable diseases.

^b^ *P* < .001.

^c^ *P* < .01

^d^ *P* < .05.

## Discussion

We observed an association between CBPs reported by parents on the RBS and subsequent self-reported insomnia symptoms in middle adulthood. To our knowledge, our study is the first to demonstrate that CBPs are consistently associated with increased risk of self-reported insomnia symptoms at 42 years of age, after accounting for important potential confounders. Furthermore, we demonstrated that these are largely associated with childhood externalizing behaviors rather than internalizing behaviors. Sleep problems during childhood (to a small extent) and mental health problems during adulthood could mediate the association between childhood behavior and insomnia symptoms during adulthood. These findings highlight the potential benefits in addressing childhood behavioral concerns, and potentially including a greater focus on sleep, to reduce adulthood insomnia. These pathways warrant much closer scrutiny to develop or inform relevant early intervention strategies.

Although a previous study demonstrated the association between sleep problems and internalizing and externalizing behaviors in adolescents,^50^ to our knowledge, no study has used a life-course approach to understand behavioral risk factors of insomnia symptoms in adulthood. A study by Dregan and Armstrong^25^ showed a positive association between adolescent sleep disturbance and adulthood sleep disturbance, after adjusting for potential confounders including behavioral problems. Furthermore, evidence shows that early-life factors, such as childhood adversity, are important factors of sleep health in adulthood.^23,27^ This finding implies that sleep health in adulthood is associated with accumulating factors throughout the life course. Childhood seems to be a critical period, particularly for insomnia.^23^ However, the pathways through which childhood behavior and adulthood insomnia are associated remain to be investigated.

We found that the risk of reported insomnia symptoms was higher for those who had externalizing behaviors, but not internalizing behaviors, at 5 and 10 years. Externalizing problems were characterized by conduct and hyperactive disorder symptoms, including fighting, lying, bullying, restlessness, irritability, and destroying belongings. In line with the current finding, children with attention-deficit/hyperactivity disorder (ADHD) are more likely to have sleep problems, including insomnia.^51,52^ A study among Australian children found an association between externalizing behavioral problems in preschool children and sleep problems in adolescence.^28^ Externalizing behaviors during childhood are more likely to persist during adulthood.^53^ It may also be that early sleep problems could be associated with emotional and behavioral problems in children.^54^ A randomized clinical trial of 244 children with ADHD demonstrated that a behavioral sleep intervention improved ADHD and conduct problem symptoms among those in the intervention group compared with those receiving usual care.^55^ Although most of these studies are limited to children and adolescents only, the findings suggest that the associations between behavioral and sleep problems are bidirectional and likely need to be addressed concurrently from early childhood. In our study, a small proportion of the association between behavioral problems at 5 years and insomnia symptoms at 42 years was mediated through childhood sleep difficulty. Thus, our finding, along with previous studies’ findings, underlines the necessity of assessing and managing both childhood sleep and behavioral problems to reduce their association with adulthood sleep health.

Although moderate and severe behavioral problems at 16 years were associated with insomnia symptoms at 42 years, externalizing and internalizing behaviors at 16 years were inversely associated with reported insomnia symptoms. This finding may be related to inaccurate parent-reported behavioral problems during late adolescence (particularly externalizing behavior^56^). Alternatively, it is possible that additional relevant factors were operating at 16 years but were not precisely measured or retained in our principal component analysis. Furthermore, the association of externalizing behaviors at 16 years with insomnia symptoms was not as consistent as the association at 5 and 10 years across different definitions, which may indicate the large amount of missing data at 16 years biasing the estimates.

This study has implications for preventive medicine. There is a consistent association of behavioral problems during childhood (particularly at ages 5 and 10 years) with insomnia symptoms in adulthood. This finding suggests that early intervention to manage CBPs, specifically externalizing behaviors, may reduce the risk of adulthood insomnia. Furthermore, the relatively small mediation effect of childhood sleep problems highlights that improving sleep outcomes in the adult population cannot be addressed solely by identifying children with sleep problems early in life. Although early sleep problems should be identified, we should additionally identify children with moderate to severe behavioral problems that persist throughout childhood as potential beneficiaries of early intervention with a sleep health focus. Given that development of children’s health beliefs has an association with engagement with, and adherence to, treatment,^57,58^ early education about the associations observed in our study may be beneficial for engaging with intervention and preventing insomnia symptoms later in life.

### Limitations

Our study has some limitations. First, standardized insomnia measures were not used in the BCS70 to measure insomnia and may result in outcome misclassification; however, the symptoms included in the current analysis reflect standardized measures and insomnia diagnostic criteria.^3^ In addition, combinations of sleep symptoms were used to define insomnia symptoms to increase the validity of insomnia classification. Furthermore, we included waking symptoms (eg, not feeling rested on waking) that could represent clinically significant insomnia symptoms.^59^ Sensitivity analysis with additional daytime symptoms did not alter our results. Second, a variable that defines insomnia associated with early termination of sleep or early morning awakenings was absent. Third, duration of insomnia symptoms was reported in the last month, unlike guidelines that advocate the use of a 3-month time frame for chronic insomnia.^39^ Fourth, there were inconsistent RBS assessment approaches at age 10 years (visual analog scale) and at ages 5 and 16 years (categorized responses), which might have resulted in heterogeneous results across the follow-up surveys. Fifth, while this is a substantially sized cohort, there has been attrition of the study sample over time, resulting in underrepresentation of certain groups (eg, men and respondents with less educated parents), although their predictive power of missingness is weak.^60^ On the other hand, it has been shown that respondents in middle adulthood (45 years of age) were in general representative of the surviving participants in the 1958 British Birth Cohort.^61^ In addition, to avoid further decline in the sample, we used multiple imputation. Sixth, in our analysis, respondents with behavioral problems could be underrepresented,^61^ resulting in attenuated association with insomnia symptoms. Seventh, uncontrolled confounding could have an effect on the estimates of association, although this is unlikely or minimal given the consistency of associations across the main and sensitivity analyses.

## Conclusions

In this population-based study, young children with severe (at ages 5 and 16 years) and externalizing (at ages 5 and 10 years) behavioral problems were associated with elevated risk of reporting insomnia symptoms in middle adulthood. Behavioral problems that persisted throughout childhood were associated with insomnia symptoms at 42 years of age. This study is the first, to our knowledge, to suggest an unfavorable association of early-life behavioral problems with adulthood sleep health, underlining the importance of treating behavioral problems in children and addressing insomnia from a life-course perspective.

## Notes

Supplement. **eAppendix.** Methods **eTable 1.** Odds Ratio (95% Confidence Intervals) for Self-Reported Difficulties Initiating or Maintain Sleep (DIMS) and at Least One Daytime Symptom (Irritability, Depression, Nervousness or Tiredness) at 42 Years of Age Associated with Behavioral Problems Measured with Rutter Behavioral Scale at 5, 10, and 16 Years of Age in the UK 1970 Birth Cohort Study **eTable 2.** Odds Ratio (95% Confidence Intervals) for Self-Reported Insomnia Symptoms at 42 Years of Age Associated with Behavioral Problems Measured with Rutter Behavioral Scale at 5, 10, and 16 Years of Age in the UK 1970 Birth Cohort Study (Childhood Sleep Difficulties, Smoking, Alcohol Consumption and Mental Well-Being were Included as Covariates) **eTable 3.** Odds Ratio (95% Confidence Intervals) for Self-Reported Insomnia Symptoms at 42 Years of Age Associated with Behavioral Problems Measured with Rutter Behavioral Scale at 5 (N = 5039), 10 (N = 4373), and 16 (N = 2300) Years of Age in the UK 1970 Birth Cohort Study (Complete Case Analysis) **eFigure 1.** Sampling Scheme (Elliott J, et al; 2016) **eFigure 2.** Direct Acyclic Graph (DAG) for the Association Between Childhood Behavioral Problems and Insomnia Symptoms at 42 Years of Age **eFigure 3.** Body Mass Index (BMI) Trajectories of Participants (10 to 42 Years of Age) in the UK 1970 Birth Cohort Study **eFigure 4.** Factor Loadings of Externalizing and Internalizing Behavioral Patterns in the UK 1970 Birth Cohort Study **eFigure 5.** Prevalence of Insomnia Symptoms at 42 Years Across Behavioral Categories and Quintiles of Externalizing and Internalizing Behavioral Problems at 5 (N = 8050), 10 (N = 9090) and 16 (N = 7653) Years of Age in the UK 1970 Birth Cohort Study
