# Supplementary material for: Association Between Childhood Behavioral Problems and Insomnia Symptoms in Adulthood
Source: JAMA Netw Open. 2019 Sep 6;2(9):e1910861. doi: 10.1001/jamanetworkopen.2019.10861 (PMC6735491; doi:10.1001/jamanetworkopen.2019.10861)
Supplement: Supplement. — eAppendix. Methods eTable 1. Odds Ratio (95% Confidence Intervals) for Self-Reported Difficulties Initiating or Maintain Sleep (DIMS) and at Least One Daytime Symptom (Irritability, Depression, Nervousness or Tiredness) at 42 Years of Age Associated with Behavioral Problems Measured with Rutter Behavioral Scale at 5, 10, and 16 Years of Age in the UK 1970 Birth Cohort Study eTable 2. Odds Ratio (95% Confidence Intervals) for Self-Reported Insomnia Symptoms at 42 Years of Age Associated with Behavioral Problems Measured with Rutter Behavioral Scale at 5, 10, and 16 Years of Age in the UK 1970 Birth Cohort Study (Childhood Sleep Difficulties, Smoking, Alcohol Consumption and Mental Well-Being were Included as Covariates) eTable 3. Odds Ratio (95% Confidence Intervals) for Self-Reported Insomnia Symptoms at 42 Years of Age Associated with Behavioral Problems Measured with Rutter Behavioral Scale at 5 (N = 5039), 10 (N = 4373), and 16 (N = 2300) Years of Age in the UK 1970 Birth Cohort Study (Complete Case Analysis) eFigure 1. Sampling Scheme (Elliott J, et al; 2016) eFigure 2. Direct Acyclic Graph (DAG) for the Association Between Childhood Behavioral Problems and Insomnia Symptoms at 42 Years of Age eFigure 3. Body Mass Index (BMI) Trajectories of Participants (10 to 42 Years of Age) in the UK 1970 Birth Cohort Study eFigure 4. Factor Loadings of Externalizing and Internalizing Behavioral Patterns in the UK 1970 Birth Cohort Study eFigure 5. Prevalence of Insomnia Symptoms at 42 Years Across Behavioral Categories and Quintiles of Externalizing and Internalizing Behavioral Problems at 5 (N = 8050), 10 (N = 9090) and 16 (N = 7653) Years of Age in the UK 1970 Birth Cohort Study [file jamanetwopen-2-e1910861-s001.pdf]

Supplementary Online Content

Melaku YA, Appleton S, Reynolds AC, et al. Association between childhood behavioral problems and insomnia symptoms in adulthood. *JAMA Netw Open.* 2019;2(9):e1910861. doi:10.1001/jamanetworkopen.2019.10861

eAppendix. Methods
eTable 1. Odds Ratio (95% Confidence Intervals) for Self-Reported Difficulties Initiating or Maintain Sleep (DIMS) and at Least One Daytime Symptom (Irritability, Depression, Nervousness or Tiredness) at 42 Years of Age Associated with Behavioral Problems Measured with Rutter Behavioral Scale at 5, 10, and 16 Years of Age in the UK 1970 Birth Cohort Study
eTable 2. Odds Ratio (95% Confidence Intervals) for Self-Reported Insomnia Symptoms at 42 Years of Age Associated with Behavioral Problems Measured with Rutter Behavioral Scale at 5, 10, and 16 Years of Age in the UK 1970 Birth Cohort Study (Childhood Sleep Difficulties, Smoking, Alcohol Consumption and Mental Well-Being were Included as Covariates)
eTable 3. Odds Ratio (95% Confidence Intervals) for Self-Reported Insomnia Symptoms at 42 Years of Age Associated with Behavioral Problems Measured with Rutter Behavioral Scale at 5 (N=5039), 10 (N= 4373), and 16 (N= 2300) Years of Age in the UK 1970 Birth Cohort Study (Complete Case Analysis)
eFigure 1. Sampling Scheme (Elliott J, et al; 2016)
eFigure 2. Direct Acyclic Graph (DAG) for the Association Between Childhood Behavioral Problems and Insomnia Symptoms AT 42 Years of Age
eFigure 3. Body Mass Index (BMI) Trajectories of Participants (10 to 42 Years of Age) in the UK 1970 Birth Cohort Study
eFigure 4. Factor Loadings of Externalizing and Internalizing Behavioral Patterns in the UK 1970 Birth Cohort Study
eFigure 5. Prevalence of Insomnia Symptoms at 42 Years Across Behavioral Categories and Quintiles of Externalizing and Internalizing Behavioral Problems at 5 (N=8050), 10 (N=9090) and 16 (N=7653) Years of Age in the UK 1970 Birth Cohort Study

This supplementary material has been provided by the authors to give readers additional information about their work.

eAppendix. Methods

Exposure

At 5, 10 and 16 years of age, parents were asked to complete a 19-item Rutter Behavior Scale questions.[1] The items are:

1. Very restless, often running about or jumping up and down
2. Is squirmy or fidgety
3. Often destroys own or others property
4. Frequently fights with other children
5. Not much liked by other children
6. Often worried, worries about many things
7. Tends to do things on own - rather solitary
8. Irritable, is quick to fly off the handle
9. Often appears miserable, unhappy, tearful or distressed
10. Sometimes takes things belonging to others
11. Has twitches, mannerisms or tics of the face or body
12. Frequently sucks thumb or fingers
13. Frequently bites nails or fingers
14. Is often disobedient
15. Cannot settle to anything for more than a few moments
16. Tends to be fearful or afraid of new things or new situations
17. Is fussy or over-particular
18. Often tells lies
19. Bullies other children

At 5 and 16 years, three responses (0=does not apply; 1=applies somewhat; 2=certainly applies) were summed up to give an overall score ranging from 0 to 38. At 10 years, a visual analogue scale system that ranges from 0 (does not apply) to 100 (certainly applies) was used.

Outcome

We used questions related to insomnia at 42 years because similar questions were not available in the previous follow ups. Questions and responses that were used to assess insomnia symptoms at 42 years are the following:

A) Difficulty initiating sleep (DIS)

During the last four weeks, how long did it usually take for you to fall asleep?

- 0-15 minutes
- 16-30 minutes
- 31-45 minutes
- 46-60 minutes
- More than 60 minutes

Those who took more than 30 minutes were considered as having DIS.

B) Difficulties maintaining sleep (DMS)

During the past four weeks, how often did you awaken during your sleep time and have trouble falling back to sleep again?

- None of the time
- A little of the time
- Some of the time
- A good bit of the time
- Most of the time
- All of the time

Those who reported a good bit of time, most of the time and all of the time were considered as having DMS.

C) Difficulties initiating or maintaining sleep (DIMS)

To define DIMS, we used DMS and DIS. Participants should have either of the two to be labelled as DIMS.

D) Not feeling rested on waking

During the past four weeks, how often did you get enough sleep to feel rested upon waking in the

© 2019 Melaku YA et al. *JAMA Network Open.*

morning?

- All of the time
- Most of the time
- good bit of the time
- Some of the time
- A little of the time
- None of the time

Participants who reported a good bit of the time, some of the time, a little of the time and none of the time were considered as not feeling rested on waking.

E) DIMS plus not feeling rested on waking (DIMS plus)

When a participant had DIMS and not felt rested on waking (DIMS plus)

Covariates

At age 5 and 16 years, data on sleep difficulty of child was collected using maternal self-completed questionnaire, with a question “Does your child have any sleeping difficulties?” and responses were “no”, “yes- mild”, “yes-severe”, yes-Not Elsewhere Classified”. We combined all “yes” responses together as a positive answer. At 10 years, parent was asked whether a child has sleep difficulty with responses of “yes” and “no”.

At 5, 10 and 16 (combined for both parents) years, maternal and paternal educational status was classified as no qualification, trade/vocational/certificate, degree and above, and other.

There were six social class categories, ranging from social class I (lowest class) to SC V (highest, professional). At 5, 10 and 16 years, we recategorized social class of mothers and fathers (combined at 5 years and separately at 10 and 16 years) into three: I & II; III non-manual & III manual and IV & V.

At 42 years, marital status was categorized into single, married/partnered, and divorced/separated/widowed. We classified social class as: I & II; III non-manual & III manual and IV & V [I, professional; II, managerial and Technical; III non manual; III manual; IV partly skilled; V unskilled]. Education qualification was categorized into five levels based on National Vocational Qualification Scale (NVQ). NVQs were work-based awards in England, Wales and Northern Ireland that given through assessment and training. We categorized smoking into non-smoker, current smoker and ex-smoker. Alcohol consumption frequency was categorized as never, monthly or less, 2-4 times a month, 2-3 times a week and 4 or more times a week. Physical activity was assessed based on frequency of different sport activities in 12 months prior to data collection and classified as never, less often, 2-3 times, once a week, 2-3 days a week, 4-5 days a week and every day.

We used body mass index from age 10, 16, 26, 30, 34 and 42 years and used group-based trajectory model [2] to assess pattern of body mass index trajectories. Body mass index was calculated from measured and self-reported weight and height. Details of data collection on BMI is described elsewhere.[3]

Perceived health status was asked at 42 years of age with response categories of “excellent”, “very good”, “Good”, “fair” and “poor”.

Statistical analysis

In the joint classification analysis, we categorized children with moderate and severe behavioral problems together.

We used “*paramed*”[4,5] command in Stata to determine the indirect effect of childhood behavioral problem on adulthood insomnia symptoms that was mediated though childhood sleep difficulty (categorical variable) and mental health assessed by the Warwick-Edinburgh Mental Wellbeing Scale (continuous). In this analysis, we determined controlled direct effect (CDI), natural indirect effect (NDI), total effect (TE) and proportion of mediation (PM). Because “*mi estimate”* is not supported by paramed, we used “*cmdok*” option in the command.

We performed multiple imputation using 30 imputed data as we did not find estimate differences beyond 30.
We combined DIMS with one of the following day time symptoms: irritability, tiredness, depression or nervousness. These symptoms are were asked using the following questions:

Tiredness: “Do you feel tired most of the time? “yes/no”

© 2019 Melaku YA et al. *JAMA Network Open.*

Irritability: ‘Are you easily upset or irritated? “yes/no”
Depressed: “Do you often feel miserable or depressed?”
“yes/no”
Nervousness: Does every little thing get on your nerves and wear you out?” “yes/no”
These questions were not asked in the context of exploring the consequences of daytime feelings of insomnia. Therefore, we did not include in the main analysis. In addition, some of these symptoms are part of the Rutter Behavioural Scale, which they could persist during adulthood and may not be related to insomnia.

© 2019 Melaku YA et al. *JAMA Network Open.*

eReferences

1. Rutter, M., Tizard, J. and Whitmore, K. Education Health and Behaviour. Longman Publishing Group, London, 1970.
2. Jones BL, Nagin DS. A Note on a Stata Plugin for Estimating Group-based Trajectory Models. *Sociological Methods & Research.* 2013;42(4):608-613.
3. Elliott J, Shepherd P. Cohort profile: 1970 British Birth Cohort (BCS70). *International journal of epidemiology.* 2006;35(4):836-843.
4. Buis ML. Direct and indirect effects in a logit model. The Stata Journal, 10(1):1129, 2010.
5. Hicks R and Tingley D. Causal Mediation Analysis. The Stata Journal, 11(4):609-15, 2011.

© 2019 Melaku YA et al. *JAMA Network Open.*

eTable 1. Odds Ratio (95% confidence intervals) for Self-Reported Difficulties Initiating or Maintain Sleep (DIMS) and at Least One Daytime Symptom (Irritability, Depression, Nervousness or Tiredness) at 42 Years of Age Associated with Behavioral Problems Measured with Rutter Behavioral Scale at 5, 10, and 16 Years of Age in the UK 1970 Birth Cohort Study

**Models**		**Odds ratio (95%**	**P for trend**	
	**Norm**	**Moderate**	**Severe**	
**Difficulties initiating or maintain sleep (DIMS) and at least one daytime**				
**5 years (N=8050)**				
Model 1	1.00	1.23(1.04-1.44)*	1.41(1.07-1.86)*	0.001
Model 3	1.00	1.14(0.96-1.36)	1.27(0.95-1.71)	0.037
**10 years**				
Model 1	1.00	1.34(1.16-1.56)***	1.31(0.95-1.82)	0.002
Model 3	1.00	1.21(1.04-1.41)*	1.08(0.74-1.57)	0.142
**16 years**				
Model 1	1.00	1.67(1.39-2.01)***	2.39(1.78-3.19)***	<0.001
Model 3	1.00	1.44(1.18-1.75)***	1.70(1.25-2.32)**	<0.001

*p<0.05; **p<0.01; ***p<0.001; **Model 1** was adjusted for sex, parents' social class, parents' education, social class, educational and marital status; **Model 2** was additionally adjusted for physical activity level, body mass index trajectory (from 10 to 42 years) , perceived health status and number of noncommunicable diseases

© 2019 Melaku YA et al. *JAMA Network Open.*

eTable 2. Odds Ratio (95% confidence intervals) for Self-Reported Insomnia Symptoms at 42 Years of Age Associated with Behavioral Problems Measured with Rutter Behavioral Scale at 5, 10, and 16 Years of Age in the UK 1970 Birth Cohort Study (Childhood Sleep Difficulties, Smoking, Alcohol Consumption and Mental Well-Being were Included as Covariates)

**Models**		**Odds ratio (95%**	**P for trend**	
	**Norm**	**Moderate**	**Severe**	
**Difficulties initiating sleep (DIS)**				
**5 years (N=8050)**				
Model 1	1.00	1.02(0.85-1.21)	1.37(1.04-1.82)*	1.00
Model 2	1.00	0.92(0.76-1.10)	1.19(0.88-1.60)	1.00
**10 years**				
Model 1	1.00	1.21(1.04-1.42)*	1.19(0.93-1.53)	0.017
Model 2	1.00	1.08(0.92-1.27)	0.92(0.71-1.21)	0.986
**16 years (**7653)				
Model 1	1.00	1.57(1.29-1.93)***	1.95(1.40-2.72)***	<0.001
Model 2	1.00	1.33(1.06-1.66)*	1.36(0.96-1.93)	0.009
**Difficulties maintaining sleep (DMS)**				
**5 years**				
Model 1	1.00	1.13(0.96-1.33)	1.21(0.90-1.62)	0.068
Model 2	1.00	1.05(0.89-1.25)	1.06(0.78-1.44)	0.528
**10 years**				
Model 1	1.00	1.19(1.01-1.40)*	1.17(0.92-1.48)	0.026
Model 2	1.00	1.07(0.91-1.27)	0.95(0.73-1.23)	0.880
**16 years**				
Model 1	1.00	1.34(1.11-1.61)**	1.95(1.40-2.71)***	<0.001
Model 2	1.00	1.16(0.95-1.42)	1.46(1.02-2.07)*	0.019
**Difficulties initiating or maintaining sleep (DIMS)**				
**5 years**				
Model 1	1.00	1.08(0.93-1.25)	1.45(1.11-1.91)**	0.009
Model 2	1.00	0.99(0.85-1.16)	1.29(0.97-1.72)	0.216
**10 years**				
Model 1	1.00	1.20(1.04-1.38)*	1.25(1.00-1.56)	0.004
Model 2	1.00	1.07(0.93-1.25)	1.00(0.78-1.29)	0.588
**16 years**				
Model 1	1.00	1.38(1.16-1.64)***	1.91(1.40-2.60)***	<0.001
Model 2	1.00	1.18(0.99-1.42)	1.40(1.01-1.94)*	0.015
**DIMS plus**				
**5 years**				
Model 1	1.00	1.08(0.91-1.27)	1.37(1.04-1.79)*	0.025
Model 2	1.00	0.98(0.83-1.17)	1.20(0.90-1.59)	0.415
**10 years**				
Model 1	1.00	1.22(1.05-1.41)**	1.33(1.05-1.68)*	0.001
Model 2	1.00	1.09(0.93-1.27)	1.08(0.83-1.39)	0.324
**16 years**				
Model 1	1.00	1.42(1.20-1.68)***	1.71(1.28-2.28)***	<0.001
Model 2	1.00	1.21(1.01-1.45)*	1.22(0.89-1.66)	0.037

*p<0.05; **p<0.01; ***p<0.001; **Model 1** was adjusted for sex, childhood sleep difficulties, parent's social class, parent's education, social class and marital status; **Model 2** was additionally adjusted for physical activity level, smoking and alcohol consumption, body mass index trajectory (from 10 to 42 years) , mental well-being, perceived health status and number of noncommunicable diseases. Imputed data were used for the analysis.

© 2019 Melaku YA et al. *JAMA Network Open.*

eTable 3. Odds Ratio (95% confidence intervals) for Self-Reported Insomnia Symptoms at 42 Years of Age Associated with Behavioral Problems Measured with Rutter Behavioral Scale at 5 (N=5039), 10 (N= 4373), and 16 (N= 2300) Years of Age in the UK 1970 Birth Cohort Study (Complete Case Analysis)

Models	-	Odds ratio (95% CI)		P for trend
	Normal	Moderate	Severe	
**Difficulties initiating sleep (DIS)**				
**5 years**				
Model 1	1.00	1.02(0.82-1.27)	1.87(1.31-2.65)***	0.016
Model 2	1.00	0.96(0.77-1.20)	1.63(1.13-2.35)**	0.025
**10 years**				
Model 1	1.00	1.25(1.01-1.55)*	1.20(0.85-1.69)	0.13
Model 2	1.00	1.09(0.87-1.36)	0.97(0.68-1.40)	0.188
**16 years (N=)**				
Model 1	1.00	1.59(1.17-2.16)**	1.52(0.95-2.44)	0.002
Model 2	1.00	1.77(1.17-2.66)**	1.36(0.69-2.65)	0.033
**Difficulties maintaining sleep (DMS)**				
**5 years**				
Model 1	1.00	1.08(0.87-1.34)	1.47(1.08-2.00)*	0.04
Model 2	1.00	1.05(0.84-1.31)	1.28(0.93-1.76)	0.06
**10 years**				
Model 1	1.00	1.39(1.14-1.69)**	0.89(0.62-1.28)	0.28
Model 2	1.00	1.25(1.01-1.54)*	0.75(0.51-1.09)	0.519
**16 years**				
Model 1	1.00	1.35(1.00-1.84)	2.10(1.37-3.22)***	0.063
Model 2	1.00	1.26(0.83-1.91)	1.33(0.69-2.55)	0.003
**Difficulties initiating or maintaining sleep (DIMS)**				
**5 years**				
Model 1	1.00	1.03(0.85-1.26)	1.81(1.37-2.39)***	0.001
Model 2	1.00	0.99(0.81-1.20)	1.57(1.18-2.10)**	0.002
**10 years**				
Model 1	1.00	1.31(1.09-1.56)**	1.09(0.81-1.48)	0.116
Model 2	1.00	1.16(0.96-1.40)	0.91(0.66-1.25)	0.158
**16 years**				
Model 1	1.00	1.58(1.21-2.06)**	1.89(1.26-2.82)**	0.002
Model 2	1.00	1.67(1.17-2.39)**	1.37(0.76-2.47)	0.001
**DIMS plus**				
**5 years**				
Model 1	1.00	1.06(0.86-1.30)	1.80(1.36-2.40)***	0.001
Model 2	1.00	1.00(0.81-1.23)	1.54(1.15-2.07)**	0.003
**10 years**				
Model 1	1.00	1.34(1.11-1.61)**	1.15(0.84-1.57)	0.06
Model 2	1.00	1.17(0.96-1.43)	0.96(0.69-1.33)	0.097
**16 years**				
Model 1	1.00	1.56(1.18-2.07)**	1.91(1.26-2.90)**	0.001
Model 2	1.00	1.74(1.19-2.54)**	1.50(0.81-2.79)	0.002

*p<0.05; **p<0.01; ***p<0.001; **Model 1** was adjusted for sex, parents' social class, parents' education status, social class, educational status, and marital status; **Model 2** was additionally adjusted for physical activity, body mass index trajectory (from 10 to 42 years), perceived health status and number of noncommunicable diseases. Analysis was conducted using complete cases)

© 2019 Melaku YA et al. *JAMA Network Open.*

eFigure 1. Sampling Scheme (Elliott J, et al; 2016)

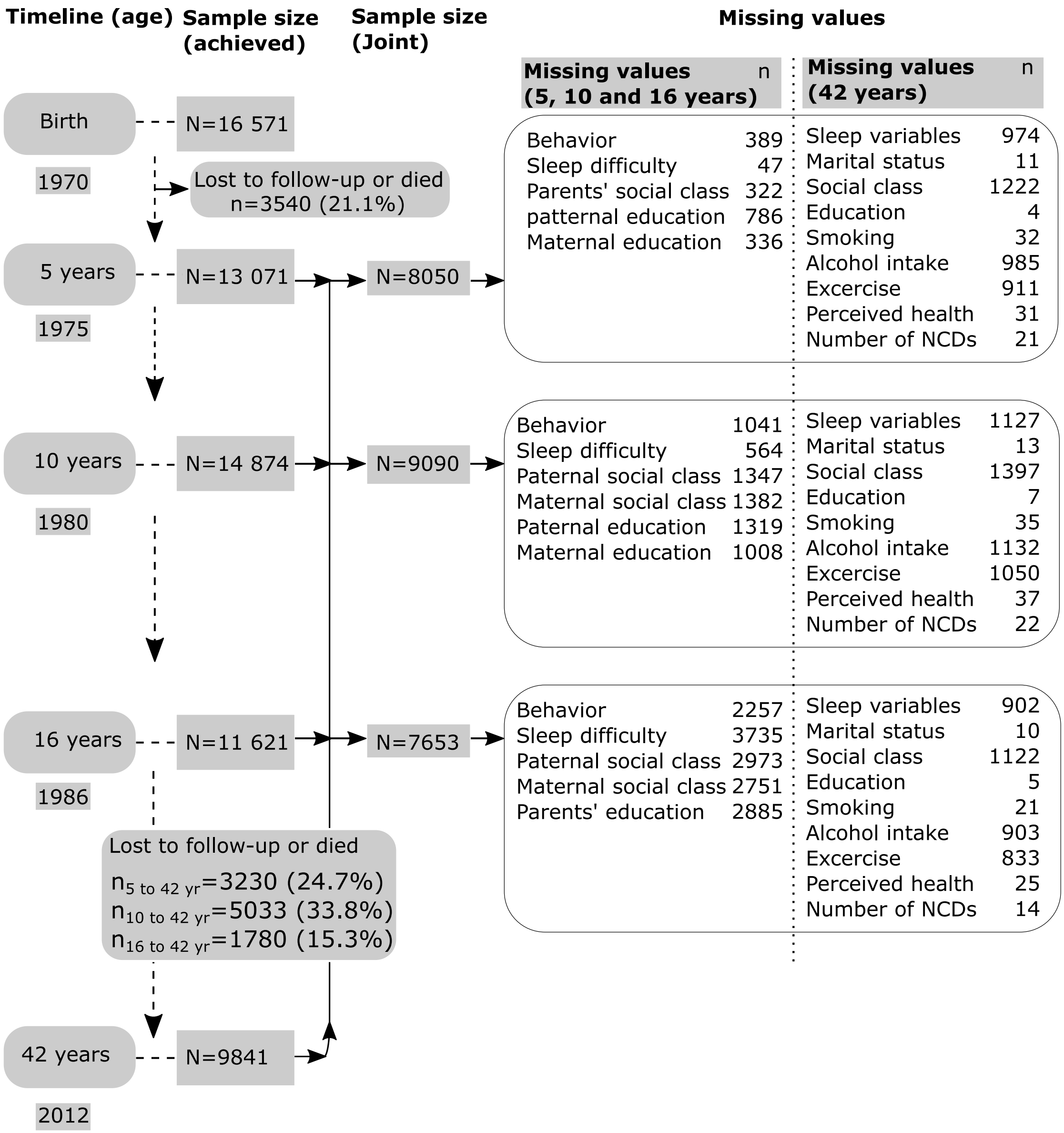

© 2019 Melaku YA et al. *JAMA Network Open.*

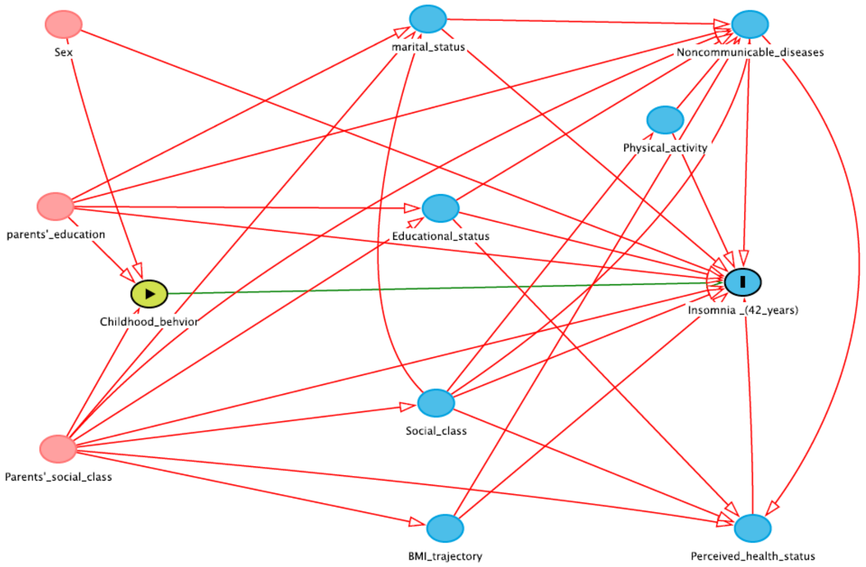

eFigure 2. Direct Acyclic Graph (DAG) for the Association Between Childhood Behavioral Problems and Insomnia Symptoms AT 42 Years of Age

© 2019 Melaku YA et al. *JAMA Network Open.*

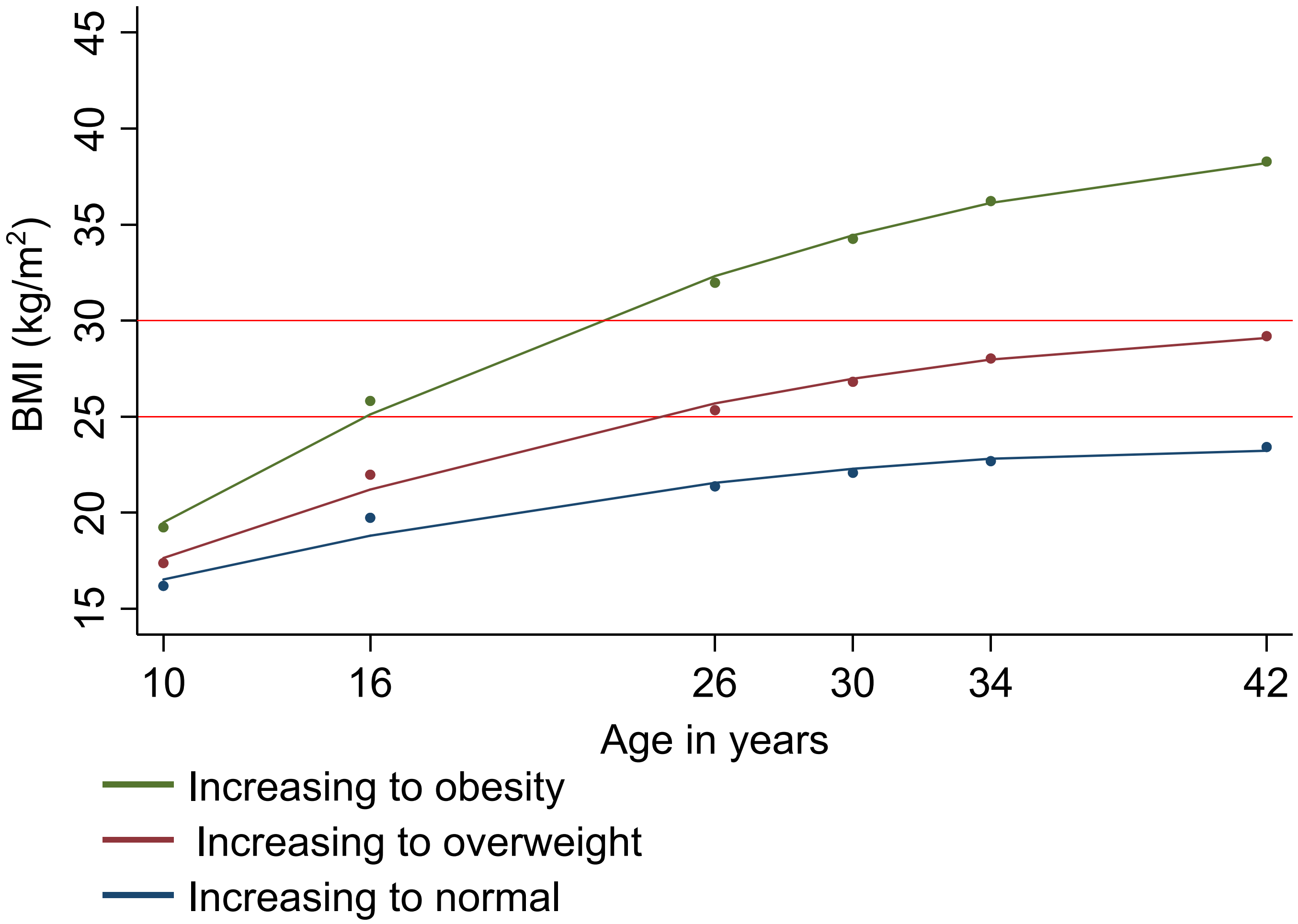

eFigure 3. Body Mass Index (BMI) Trajectories of Participants (10 to 42 Years of Age) in the UK 1970 Birth Cohort Study [*Figure is based on observed data]*

© 2019 Melaku YA et al. *JAMA Network Open.*

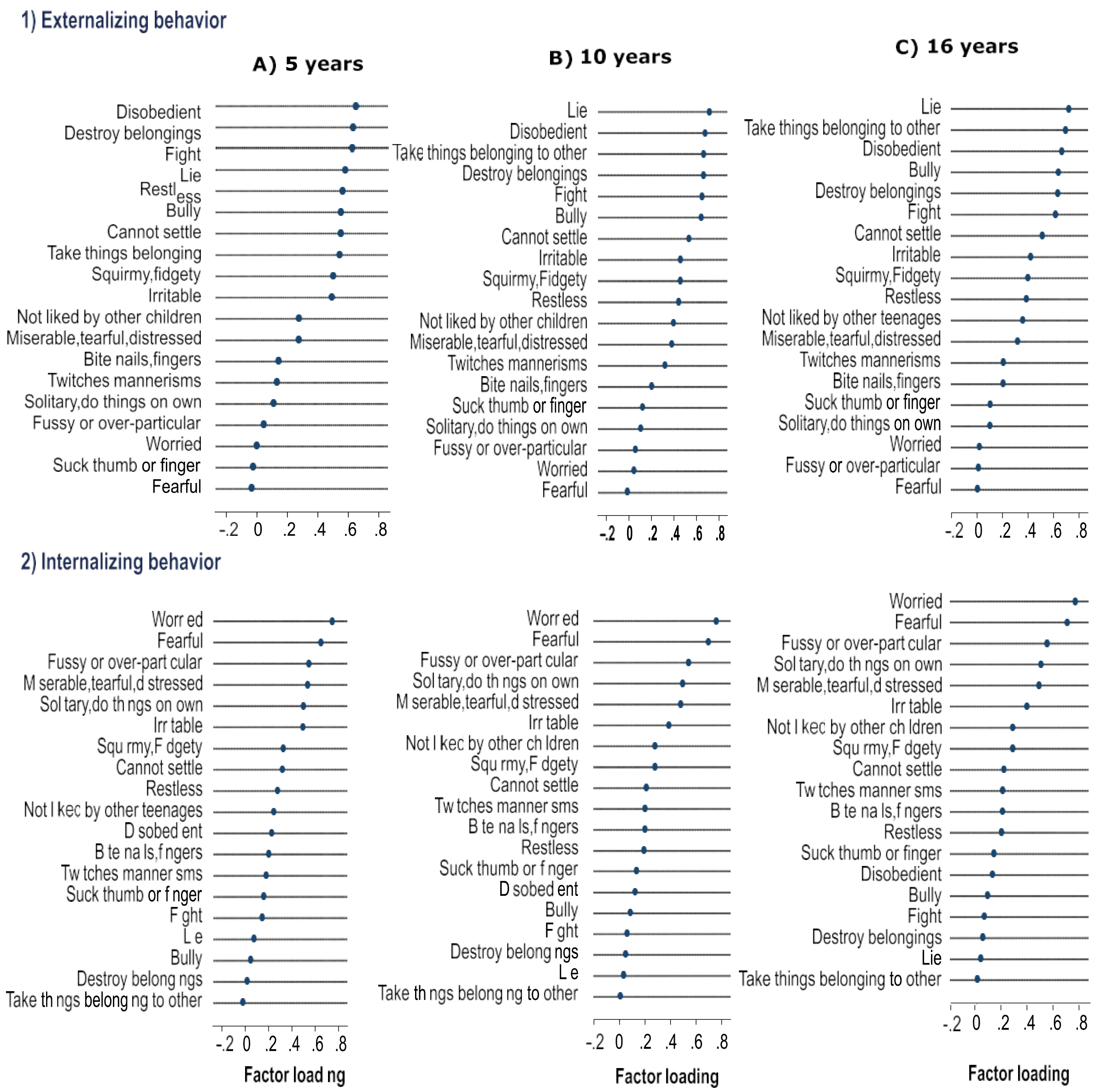

eFigure 4. Factor Loadings of Externalizing and Internalizing Behavioral Patterns in the UK 1970 Birth Cohort Study (Figure is based on observed data.)

© 2019 Melaku YA et al. *JAMA Network Open.*

eFigure 5. Prevalence of Insomnia Symptoms at 42 Years Across Behavioral Categories and Quintiles of Externalizing and Internalizing Behavioral Problems at 5 (N=8050), 10 (N=9090) and 16 (N=7653) Years of Age in the UK 1970 Birth Cohort Study (Figure is based on imputed data)

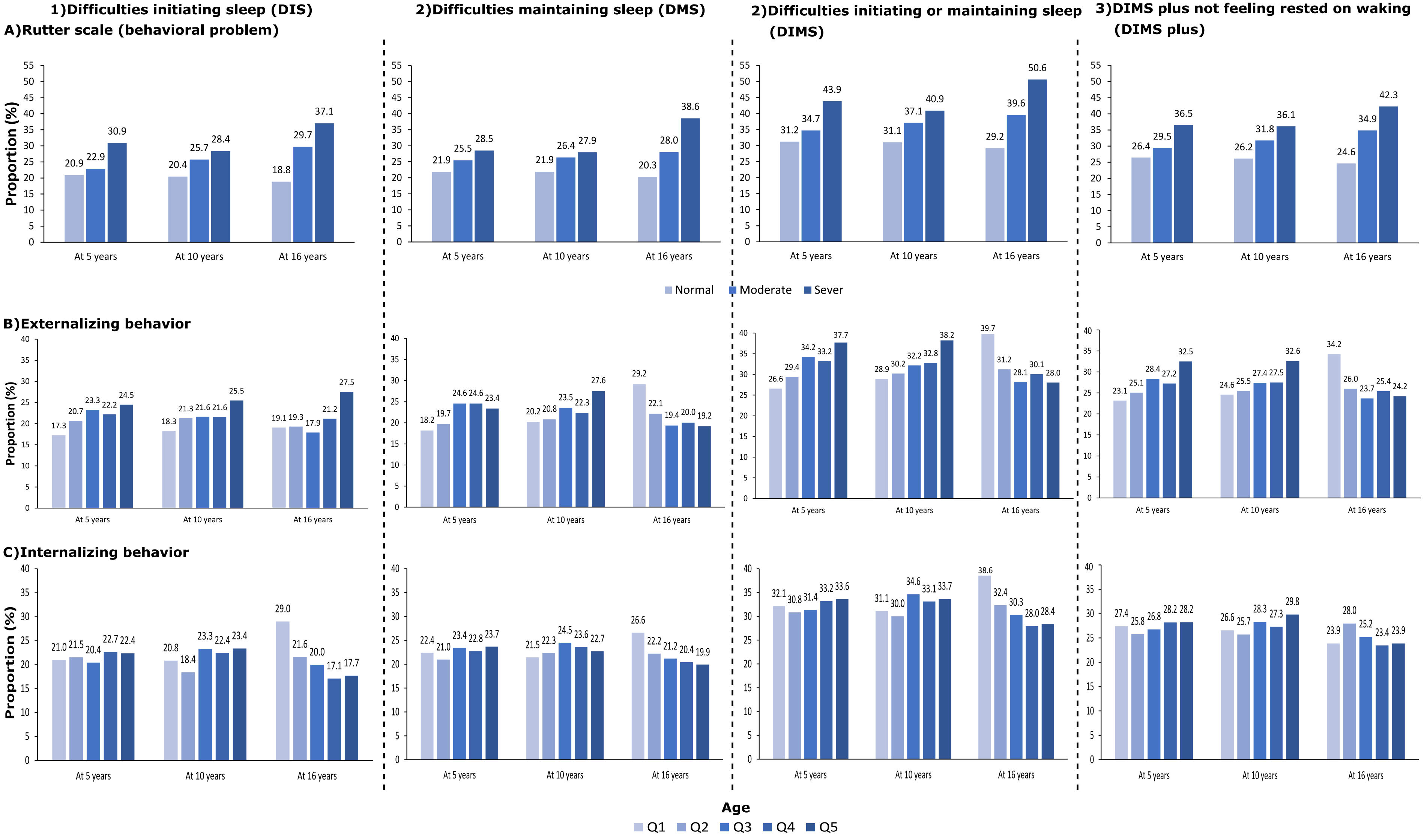

© 2019 Melaku YA et al. *JAMA Network Open*.
